# The Influence of Between-Farm Distance and Farm Size on the Spread of Classical Swine Fever during the 1997–1998 Epidemic in The Netherlands

**DOI:** 10.1371/journal.pone.0095278

**Published:** 2014-04-18

**Authors:** Gert Jan Boender, Rob van den Hengel, Herman J. W. van. Roermund, Thomas J. Hagenaars

**Affiliations:** Department of Epidemiology, Crisis organization and Diagnostics, Central Veterinary Institute (CVI) of Wageningen, Lelystad, The Netherlands; Auburn University, United States of America

## Abstract

As the size of livestock farms in The Netherlands is on the increase for economic reasons, an important question is how disease introduction risks and risks of onward transmission scale with farm size (i.e. with the number of animals on the farm). Here we use the epidemic data of the 1997–1998 epidemic of Classical Swine Fever (CSF) Virus in The Netherlands to address this question for CSF risks. This dataset is one of the most powerful ones statistically as in this epidemic a total of 428 pig farms where infected, with the majority of farm sizes ranging between 27 and 1750 pigs, including piglets. We have extended the earlier models for the transmission risk as a function of between-farm distance, by adding two factors. These factors describe the effect of farm size on the susceptibility of a ‘receiving’ farm and on the infectivity of a ‘sending’ farm (or ‘source’ farm), respectively. Using the best-fitting model, we show that the size of a farm has a significant influence on both farm-level susceptibility and infectivity for CSF. Although larger farms are both more susceptible to CSF and, when infected, more infectious to other farms than smaller farms, the increase is less than linear. The higher the farm size, the smaller the effect of increments of farm size on the susceptibility and infectivity of a farm. Because of changes in the Dutch pig farming characteristics, a straightforward extrapolation of the observed farm size dependencies from 1997/1998 to present times would not be justified. However, based on our results one may expect that also for the current pig farming characteristics in The Netherlands, farm susceptibility and infectivity depend non-linearly on farm size, with some saturation effect for relatively large farm sizes.

## Introduction

During the last two decades the mean size of pig herds has increased in many European countries, including The Netherlands. In 1997 around 14.5 million pigs were present in The Netherlands, housed in 21,500 farms, leading to an average number of 674 pigs (including piglets) per farm [1]. In 2000 the population size had declined to around 13.1 million pigs, distributed over about 14.500 farms, and in 2010 this was around 12.3 million pigs on about 7.000 farms [2]. So the average farm size increased from 903 pigs in 2000 to 1743 in 2010, and the number of pig farms decreased from 14,500 to 7,000, thus increasing the average between-farm distance. There is a public debate about the desirability of large farms, concerning animal welfare and disease aspects. For this discussion about the advantages and disadvantages of large farms, it is important to know the consequences of a large farm size for the risk of epidemics of contagious animal diseases.

In 1997–1998 an epidemic of classical swine fever (CSF) occurred in The Netherlands. During the entire epidemic, 428 pig farms were infected (out of a total of 21,500 farms in The Netherlands) and 1286 farms were culled preemptively [1], [3]–[5]. In this epidemic, even after movement restrictions and enhanced biosecurity measures were implemented (as a response to the detection of the first outbreak farm), the between-farm CSF transmission continued for considerable time. This continuing transmission must have been at least in part due to indirect contacts still occurring between farms [6]. Previous analysis of the between-farm transmission of CSF in this epidemic has illustrated that for a major part of the infected farms, no contact to a previously infected farm was traced [6], [7]. This implies that the full observed between-farm transmission cannot be described by a model of known indirect contacts. In order to still quantitatively describe the (full) between-farm transmission we therefore model all transmission routes together by one distance-dependent phenomenological probability function [7], [8]. This so-called transmission kernel describes the probability of transmission of CSF as a function of the distance between ‘receiving’ and ‘source’ farm, either per day or accumulated over the infectious period of the ‘source’ farm. The first estimate of the transmission kernel, in this case restricted to the transmission risks of untraced contacts, was given by Stegeman et al. [6] assuming a piecewise constant function with a maximum transmission distance of 2 km. An estimation of a smooth parameterized transmission kernel, now applying to all possible contacts together and extending beyond 2 km, was reported in the paper by Backer et al. [9]. According to both kernels, the probability of a susceptible farm to get infected by a ‘source’ farm decreased with increasing distance to that ‘source’ farm. In both studies the transmission kernel is formulated independently of farm size (number of pigs per farm). In the present study we extend the earlier model (kernel) for the transmission risk as a function of between-farm distance, by adding two factors. These factors describe the farm-size dependence of, respectively, the susceptibility of an ‘infection-receiving’ farm (exposed farm) and the infectivity of an infected or ‘source’ farm. The Dutch classical swine fever (CSF) dataset is one of the most powerful ones statistically to study these farm-size dependencies, the drawback being that it applies to the situation of 15 years ago so that outcomes of the analysis cannot be straightforwardly extrapolated to current times.

## Methods

### Data selection

As is explained in the ‘Data analysis’ section below, our analysis requires the following pieces of information: the geographical locations of all pig farms at the time of the epidemic, the number of animals on each farm, the day of infection and day of culling of each detected outbreak farm, and the day of culling of each preemptively culled farm.

#### Pig farms

The locations of all pig farms in The Netherlands at the time of the epidemic and the number of pigs per farm (or more precisely, the maximum number of pigs allowed at that particular location by the production license of the farm) were obtained using a database from the Dutch Animal Health Service (AHS). For a description of this database, see Jalvingh et al. [10]. When a farm had pigs on more than one location (this occurred for only 0.1% of farms), we assumed that the number of pigs was divided equally over the different locations; each location was taken into account as a separate unit in the analysis. When one location was shared by more than one farm (i.e. farm license), it was considered as one single farm with a number of pigs given by the sum of the numbers of pigs in the farms (licenses) sharing the location. For breeding farms the number of pigs in the database did not include piglets. To obtain the total number of animals per farm the number of piglets was estimated and added to the number of sows. The number of piglets was obtained by multiplying the number of sows by 4.4, based on the fact that on average at any moment during the production cycle a sow has approximately 4.4 piglets [11].

For four outbreak farms the production license data indicated that the license did not allow pig production on that location, i.e. the farm license data suggested these were “empty farms”; in these cases we used the number of pigs noted in the outbreak inspection report to avoid inconsistencies in our analysis. All non-outbreak empty farms were removed from the dataset. After these corrections, the dataset contained 23,131 pig farms, with farm size ranging from 1 to 21,740 animals, with median 400 and mean *N^–^* = 657.3. The size of the outbreak farms ranged between 1 and 21,740, with median 1296.5 and mean *N^–^* = 1516.4. Thus, larger farms are overrepresented in the set of outbreak farms. For more basic characteristics of the data base, see Table 1. During the first phase of the 1997–1998 epidemic an artificial insemination (AI) station became infected, and for 21 subsequent outbreak farms the CSF infection by means of artificial insemination (AI) was regarded as a possible transmission route [12]. In order to take into account the AI station as infection source in our analysis, we added the location of the infected AI station to the location data and assigned a fictitious ‘effective’ farm size of 3225 animals to this location. This number was calculated based on assuming that farm infectivity scales linearly proportionally with farm size. The rationale for this choice is to avoid that the AI station would introduce any bias in the data towards other than the simplest possible (namely linear) size dependence. The calculation of the effective farm size went as follows: we multiply the average farm size in the outbreak area (1038 animals) with the ratio between the observed number of secondary farm infections caused by the AI station (21) and the observed average number of secondary farm infections caused by one infectious farm during the first phase of the outbreak (this is given by the between-farm *R*_0_ which was 6.76 as estimated by Stegeman et al. [7]). For a precise definition of the ‘outbreak area’ we refer to ‘Computational method’.

**Table 1:** Data characteristics of the pig population in the Netherland during the 1997/1998 CSF outbreak.

	All farms	All infected farms	Farms in OA	Infected farms in OA
#	23131	428	5396	406
*_N_^–^*	657.3	1516.4	1038.3	1515.7
min	1	1	1	1
Q_1_	160	848	360	848
Median	400	1296.5	806.9	1285.2
Q_3_	901	1800	1375	1800
max	21740	21740	21740	21740

# is number of farms, 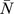
is the average farm size; the farm-size distribution is characterized by five quantities: *min* is the minimum farm size observed in the dataset, Q1 the lower quartile, Q3 the upper quartile, and *max* the maximum farm size observed. OA is the Outbreak Area (see Figure 1).

#### Infection moment

For the day of infection of each infected farm, we used the estimates obtained by Stegeman et al. [7]. We assumed that an infected farm becomes infectious 7 days after infection, and that the infectious period ends on the day of culling of the farm.

#### Pre-emptive culling of pig farms

We had no access to data on the location coordinates and culling dates of individual farms that were preemptively culled. We therefore approximately reconstructed these data from available information on preventive ring culling measures, which started on 20 April 1997, as follows. All farms within a 1 km radius of a detected outbreak farm were assumed to have been preemptively culled either 4days after detection and culling of the corresponding outbreak farm or on 20 April, whichever date was the latest. The 4-day delay is the median delay of preemptive culling according to Elbers et al. [4].

### Data analysis

The goal of our analysis is to determine both the distance-dependence and the farm-size dependence of the between-farm transmission risk of CSF during the Dutch 1997/1998 epidemic. We will do this by describing the transmission hazard between an infected pig farm and a susceptible pig farm in terms of the distance between the two farms and the sizes of both farms using a transmission kernel, and then use maximum-likelihood estimation to determine the parameters of this kernel. For the farm-size dependence we will use a set of six different candidate parameterizations in order to describe a broad range of possible dependencies on farm size (including farm-size *in*dependence). This allows us to identify a best-fitting size dependence parameterization using Akaike' Information Criterion (AIC) [13]. For the distance-dependence we use a parameterization that presumes a risk that declines with distance in accordance with previous findings [6], [9].

#### Transmission kernel

We quantify the transmission between an infected pig farm of size 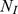
and a susceptible farm of size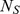 by means of the following statistical model:
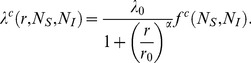
(1)Here *λ^c^* is the ‘transmission kernel’ defined as the between-farm transmission probability per unit of time as a function of *r*, *N_s_* and *N_i_*, where *r* is the between-farm distance. Here λ_0_ is the transmission probability per unit of time at distance zero, *r*_0_ is the distance for which the rate is half λ_0_, α is the power which determines the slope at which the transmission rate decreases as a function of distance, and 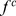 describes how the transmission probability is influenced by farm size: 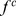
is depending on the number of animals on the susceptible farm 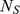
and on the number of animals on the infectious farm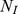. Different candidate kernel parameterizations have been considered in this study, where *c* stands for the candidate number:
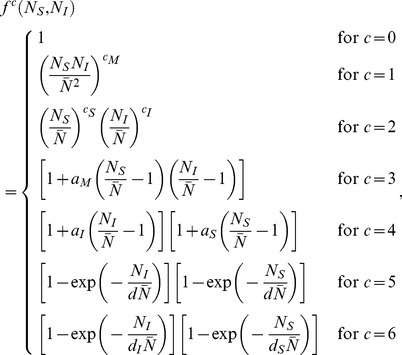
(2)in which the exponents 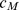, 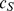 and 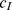, the coefficients 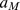, 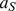 and 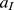, and the scaling parameters 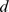, 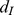 and 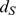 are parameters to be estimated, and 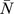 is the mean farm size. Together these candidate parameterizations describe a broad range of possible dependencies on farm size. 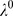, i.e. the kernel for *c = *0 is the farm-size independent kernel as used by Boender et al. [14]. The parameterizations *c = *1 and *c = *2 assume a power-law dependence on farm size, with *c = *1 using the same exponent 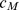 for both farm susceptibility and infectivity and with *c = *2 accommodating the possibility of estimating different exponents for susceptibility and infectivity. The parameterization *c = *3 assumes that the difference from the mean farm-size value of the kernel is a product of linear dependencies on 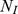 and on 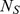. *c = *4 assumes a linear dependence of both susceptibility and infectivity on farm size. The last two candidates (*c = *5 and *c = *6) are equivalent to the kernel parameterization used by Chis Ster et al. [15] in an analysis of between-farm transmission during the 2001 foot-and-mouth disease epidemic in Great Britain. Candidate *c = *5 has only one scaling parameter 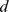, and *c = *6 has different scaling parameters 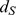 and 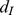 for respectively farm susceptibility and infectivity. The farm-size dependencies in *c = *5 and *c = *6 have a maximum (‘ceiling’) of 1, and for small sizes they reduce in good approximation to a linear dependence, as a first-order Taylor expansion in 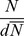
shows: 
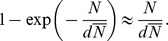
From this result we see that for low farm sizes the product 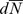 can be interpreted as an inverse proportionality constant relating farm susceptibility/infectivity to farm size. We note that the form of Eq. (1) for the transmission kernel falls in the general class of ‘gravity models’ as discussed e.g. in [16], [17].

#### Parameter estimation method

Parameter estimates are obtained using maximum-likelihood estimation. The likelihood is given by the following expression:
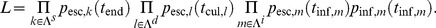
(3)The quantity 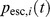
is the probability that farm *i* is escaping from infection (by any of the infectious farms) up to time *t* and the quantity 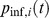 is the probability that farm *i* is infected by any of the infectious farms at time *t*. Here the total number of farms is subdivided in three sets: Λ^i^ is the set of all farms that are infected by the disease during the epidemic, Λ^d^ is the set of all preemptively culled farms, and Λ^s^ is the set of all farms remaining susceptible. Any farm *i* from the set Λ^i^ escapes from infection until it is infected at time *t*_inf,*i*_, any farm *i* from the set Λ^d^ escapes until it is culled at time *t*_cul,*i*_, and any farm *i* from the set Λ^s^ escapes until the end time *t*_end_ of the experiment. The escape probability 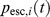
is given by
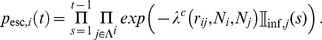
(4)Here 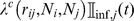 is the probability per day that an infectious farm *j* infects a susceptible farm *i* at time *t*, where *r_ij_* is the distance between farms *i* and *j*, *N_i_* and *N_j_* are the number of animals on farms *i* and *j* respectively and 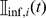
denotes the indicator function which is 1 when farm *j* is infectious at time *t*, and 0 otherwise. The infection probability 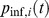 is given by
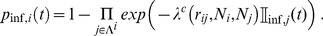
(5)For a complete dataset, which contains the geographical location coordinates of all pig farms in The Netherlands, the number of animals on each farm during the epidemic, the estimated day of infection of each outbreak farm, and the culling day of each infected farm and preemptively culled farm, we can calculate the likelihood and thus estimate the parameters *λ*_0_, *r*_0_, *α* and the *k*-3 parameters determining *f_c_*, with *k* the total degrees of freedom.

#### Computational method

**Figure 1:**
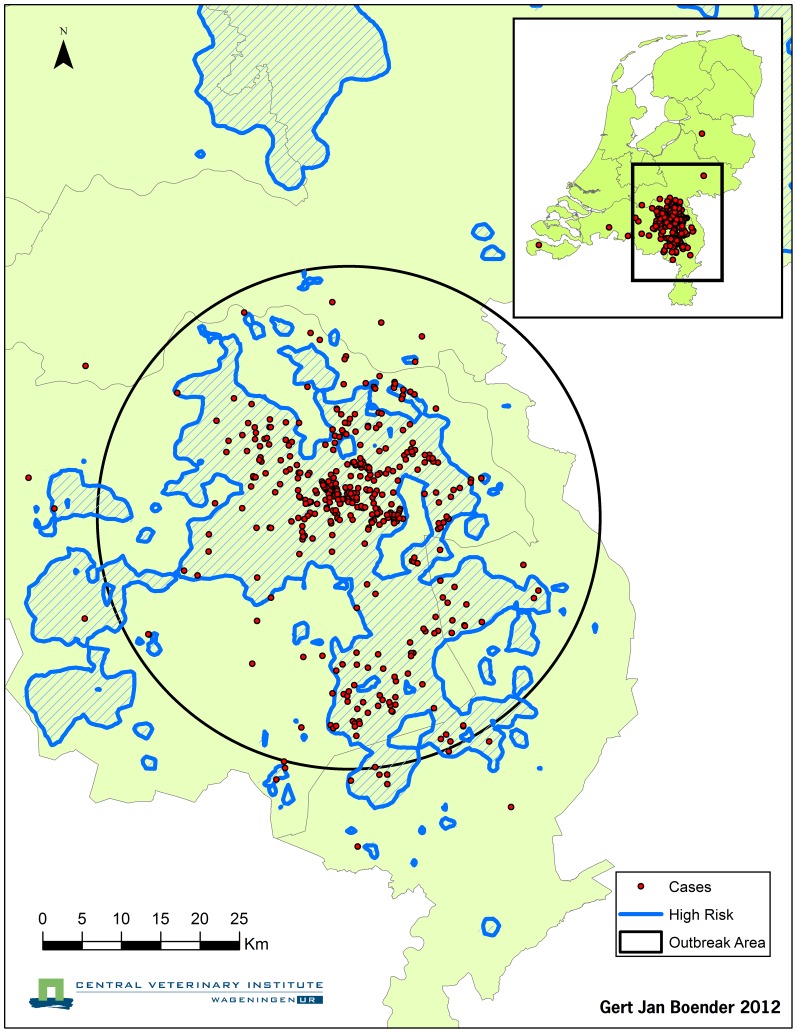
Map with the Outbreak Area (OA, black circle) in The Netherlands. This includes the infected farms (red dots) and the high-risk areas (blue). The high-risk areas for transmission of CSF (blue) were calculated using the basic kernel (without farm-size dependence), using the method of Boender et al. [8], [20].

Numerical optimization of the likelihood across all 7 candidate parameterizations *f_c_* proved prohibitively time consuming when using the full dataset with *all* pig farms in The Netherlands. We therefore reduced the computational burden by approximating the full likelihood by one in which only the farms within the circular ‘outbreak area’ (OA) in The Netherlands are included. All farms outside the OA are assumed to escape infection, and these escapes are taken into account in the likelihood in an approximate fashion. The radius of the OA (31.9 km) was determined such that the OA contained at least 95% of all outbreak farms, and the coordinates of its center were calculated by taking the mean over all x-coordinates and over all y-coordinates of all outbreak farms. The characteristics of the OA are presented in Table 1 and the OA is visualized in Figure 1. The contribution to the likelihood of the escape from infection of all farms outside the OA was approximated by modeling the region of The Netherlands outside the OA area as half a ring (with an outer radius of 200 km and inner radius of 31.9 km) with a uniform pig farm density 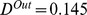
farms/km^2^ such that the total number of farms matches the number of pig farms in The Netherlands outside the OA. This contribution then takes the form
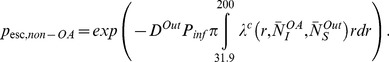
(6)Here, 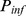 is the sum of the infectious-period lengths of all outbreak farms, 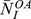 is the average farm size of infectious farms inside the OA (which is 799.1), while 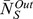 is the average farm size of susceptible farms outside the OA (which is 345.5).

Using this approximation, we compared the Maximum Likelihood for all candidate kernel parameterizations presented in Equation (1) and (2) using Akaike's Information Criterion (AIC) [13]. For the kernels with the lowest AIC (i.e. kernels with the best fit), the maximum-likelihood estimation was then also carried out with the full dataset taking into account the individual locations of all pig farms in The Netherlands. In the approximated dataset analysis we are mainly interested in comparing AICs, and we therefore only calculate parameter point estimates and no confidence bounds. In the maximum-likelihood estimation of the best-fit models using the full dataset we also calculate parameter confidence bounds.

## Results

**Figure 2:**
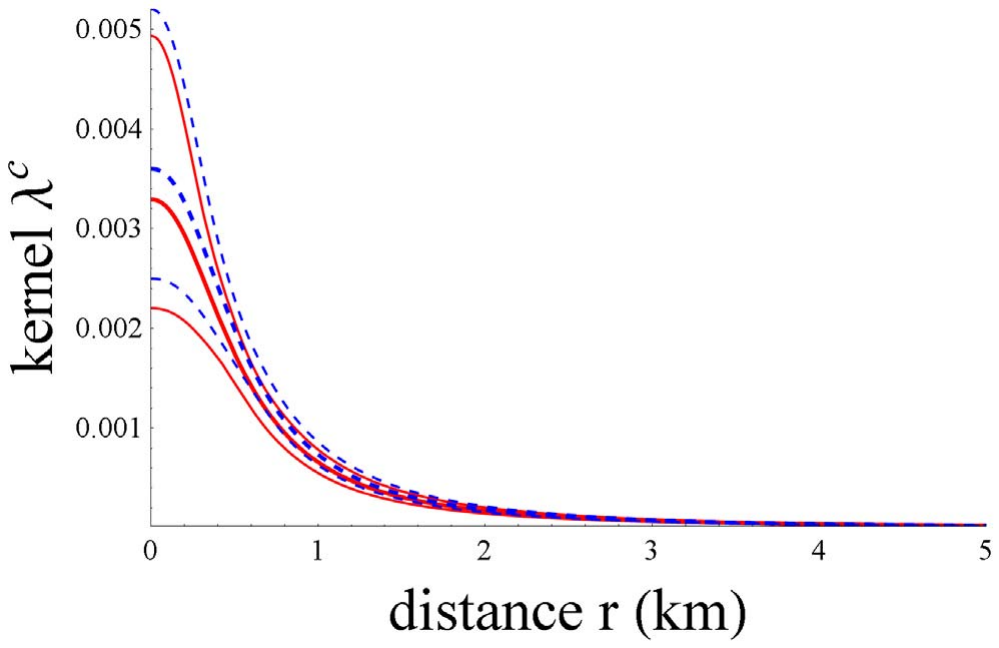
Estimated transmission kernels λ^c^ and their confidence bounds. The basic kernel parameterization is given by *c = *0 in Equation (1) and (2) without farm-size dependence (dashed blue line) and the best-fit kernel *c = *5 (solid red line), where N_S_ is set equal to the average size of the farms in the OA (1038.3) and N_I_ to the average size of the infected farms in the OA (1515.7), with their confidence bounds (thinner lines).

In Table 1 the farm-size characteristics of the Dutch pig population during the 1997/1998 CSF epidemic is shown for infectious and susceptible farms in both the OA and The Netherlands at large. The likelihood is maximized for the OA for each of the 7 candidate parameterizations presented in Equation (1) and (2). The performances of the different candidates are given in Table 2. According to this table, candidate *c = *5 has the lowest AIC and thus the best fit and therefore gives the best description of the OA data. In particular, we find that assuming farm-size independence (*c = *0) leads to a comparatively poor fit. The best description *c = *5, with the estimated parameter values of Table 2, confirms that between-farm transmission decreases with increasing between-farm distance (see Figure 2). Furthermore, between-farm transmission is found to be increased both by a higher number of animals on the susceptible farm and by a higher number of animals on the infectious farm. This increase is non-linear: it is proportional to farm size for relatively low sizes, and for higher sizes it levels off until reaching a ceiling at about 3500 animals per farm (for derivation see below). We note that the fit of *c = *1 is not significantly different from that of *c = *5, as the difference between the two AIC's is smaller than 2 (ΔAIC<2.0) [18]. The shape of the *c = *1 model curve, with the estimated parameter values of Table 2, is very similar to that of *c = *5 as described above.

**Table 2:** The AIC values (Akaike' Information Criterion, see [13]) for the candidate kernel parameterizations for the OA dataset.

	Optimal spatial parameter values			Optimal farm-size parameter values		
*c*	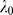 (day^−1^)	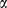	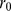 (km)		*k*	AIC
0	0.0027	2.12	0.46		3	6489.66
1	0.0024	2.08	0.43	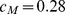	4	6452.66
2	0.0024	2.1	0.45	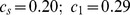	5	6454.60
3	0.0026	2.09	0.44	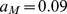	4	6483.48
4	0.0024	2.10	0.44	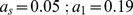	5	6471.24
5	0.0040	2.10	0.46	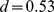	4	6451.24
6	0.0042	2.10	0.45	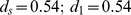	5	6453.24

A lower AIC value corresponds to a better fit to the data. For explanation of the parameters, see Equation (1) and (2). *k* is the total degrees of freedom.

Candidates *c = *2 and *c = *6 are extensions of *c = *1 and *c = *5, respectively. Splitting of the mean parameter (in candidates 1 and 5) into separate parameters for the susceptible and for the infectious farms (in candidates 2 and 6) does not lead to a significantly better fit with clearly distinct parameter values for both types of farms. Therefore, candidates *c = *2 and *c = *6 can be disregarded. The candidates *c = *3 and *c = *4 yield significantly worse fits (ΔAIC>2.0) than *c = *1 and *c = *5 and are therefore also disregarded from now on.

**Figure 3:**
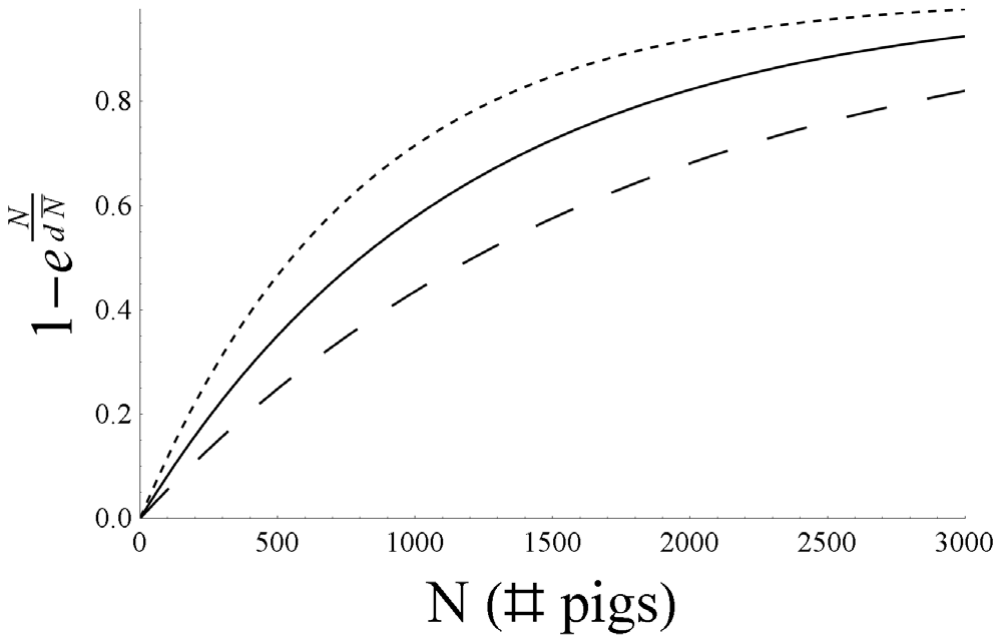
The relative susceptibility or infectivity of a farm as a function of its size, according to the best-fit parameterization *c = *5 (maximum susceptibility or infectivity equals 1). Full line: point estimate (d = 1.76), short-dashed line: lower bound (d = 1.21), long-dashed line: upper bound (d = 2.66).

**Figure 4:**
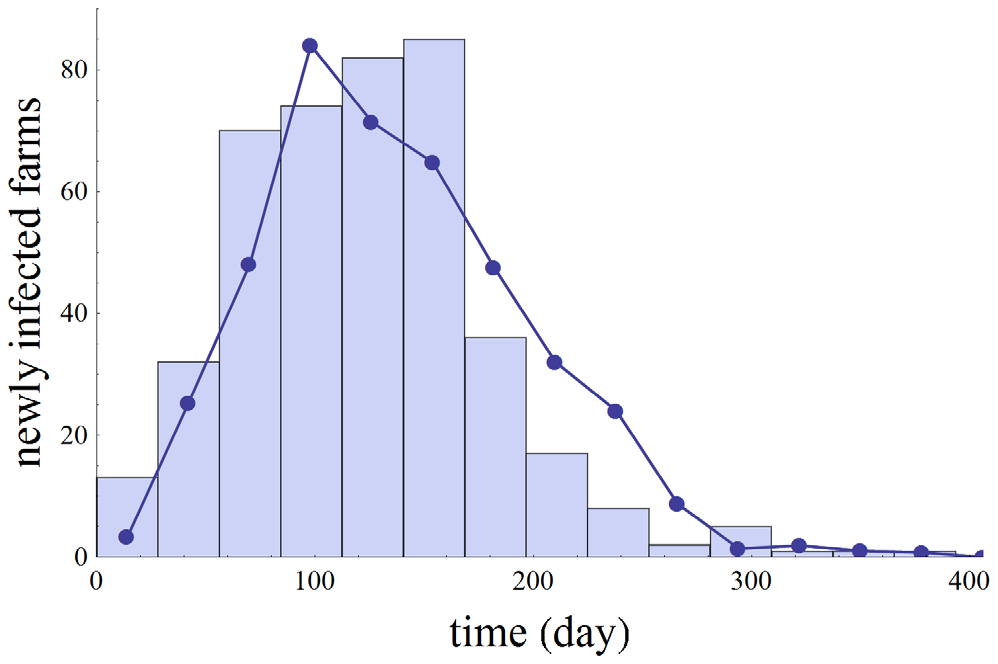
Comparison of the best-fit model prediction to the observed epidemic in 1997/1998. Number of newly infected farms per 28-day period: as derived directly from the 1997/1998 CSF epidemic data (bars) and as predicted by the fitted *c = *5 model for the between-farm transmission risk (line with symbols). Here time t = 0 corresponds to 24 December 1996.

For the best candidate parameterizations (*c = *1 and *c = *5) we now perform maximum-likelihood parameter estimation using the *full* dataset. In Table 3 the results are presented and compared with the full-data maximum-likelihood estimation result for the basic kernel without farm-size dependence, i.e. *c = *0. For the total dataset, parameterization *c = *5 gives a significantly better fit than *c = *1 (ΔAIC>2.0). For this reason, we continue with *c = *5 and compare it with *c = *0. In Figure 2 both kernels are depicted as a function of the between-farm distance. Here 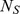 is set equal to that of the farms in the OA (1038.3) and 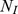 to that of the infected farms in the OA (1515.7), according to Table 1. Furthermore, the estimated values for λ_0_, r_0_, α and d, with their 95%-confidence intervals, are used. According to Equation (1) and (2) 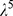 has a ceiling for large farm sizes. In Figure 3 the farm-size dependence of the farm susceptibility or infectivity for parameterization *c = *5, i.e. 
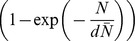
, is plotted for the point estimate, the lower bound and the upper bound of *d*. For the estimated value d = 1.76, this expression is reaching the 95% level of its ceiling for a farm size of about 3500. In Figure 4 we compare the observed monthly number of new outbreak farms to the *c = *5 model prediction.

**Table 3:** AIC values (Akaike's Information Criterion, see [13]) and parameter estimations for candidate kernel parameterizations for the *full* dataset.

c	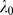 (day^−1^)	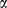	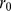 (km)	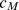 or d	AIC
0	0.0036 (0.0025–0.0053)	2.27 (2.15–2.40)	0.55 (0.42–0.73)		6801.94
1	0.002 (0.0014–0.003)	2.18 (2.08–2.32)	0.51 (0.39–0.69)	0.54 (0.46–0.64)	6681.14
5	0.0104 (0.0060–0.0195)	2.18 (2.06–2.32)	0.53 (0.38–0.72)	1.76 (1.21–2.66)	6665.84

The calculations were performed for the basic kernel without farm-size dependence (*c = *0) and the two best-fitting candidates *c = *1 and *c = *5. A lower AIC value corresponds to a better fit to the data. For explanation of the parameters, see Equation (1) and (2).

## Discussion

The goal of our analysis was to determine both the distance-dependence and the farm-size dependence of the between-farm transmission risk of CSF during the Dutch 1997/1998 epidemic. We did this by describing the transmission hazard between an infected farm and a susceptible farm in terms of the distance between the two farms and the sizes of both farms, using 7 candidate transmission kernel parameterizations (see Equation 2), and then used maximum-likelihood estimation to determine the parameters of these parameterizations.

An important conclusion of our analysis is that the number of pigs on the farms influences the between-farm transmission risk: the larger the farms, the higher the transmission risk. Our analysis further confirms that between-farm distance has an important influence on transmission risk, as was already concluded in previous work: the longer the distance, the lower the transmission risk [6], [19]. The best fitting model *c = *5 implies that a larger farm size increases both the susceptibility of the ‘receiving’ farm and the infectivity of the ‘source’ farm, without making a difference between the strength of these two effects (one and the same parameter *d* in Equation 2 for both effects). When using a parameterization that does distinguish between the farm-size dependence of susceptibility and that of infectivity (*c = *6), no significant difference between the parameter values was found. We note that the *distance*-dependence within the basic kernel (*c = *0) and the best kernel (*c = *5) are statistically indistinguishable, so the decrease of between-farm transmission with increasing between-farm distance is the same, i.e. it is not affected by the inclusion of farm-size dependence in the analysis.

We showed that the size of a farm has a significant influence on both farm-level susceptibility and infectivity for CSF (see Figure 3). Although larger farms are both more susceptible to CSF and, when infected, more infectious to other farms than smaller farms, for each of these effects the increase is weaker than proportional to farm size. The effect of farm size becomes less noticeable for larger farm sizes, there is a ‘saturation effect’, i.e. susceptibility and infectivity become almost size-independent. However, we note that the slope of the farm-size dependence for large farm sizes is subject to high statistical uncertainty, because of the limited number of large (>4000 pigs per farm) farms in the Dutch CSF database. For the infectivity of the ‘source’ farms the saturation effect can be explained as follows. As the detection is becoming highly likely once the number of animals with clinical signs exceeds a certain value, the number of infectious animals at the moment of detection of the farm (and subsequent cull) is expected to become independent of farm size for farm sizes above a certain value. Assuming that the (maximum) relative infectivity of farms is determined by the number of infectious animals (at the moment of detection) on the farms, this would explain the saturation effect. An implication of this explanation that is important for extrapolation purposes is that the farm-size dependence estimated here will remain unaffected when the mean farm size 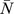
changes; more precisely, the implication is that the product 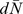
will remain unaffected by changes in 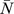.

Since 1997 there has been a continuous trend in the Dutch pig farm structure towards a lower number of farms and a higher average farm size (with the total number of pigs in the country remaining similar). The net effect on the between-farm transmission risks during a CSF epidemic (in the presence of movement restrictions) is not straightforward. We can use the distance and farm-size dependencies estimated here to derive some insight into the effect of these changes on the between-farm CSF transmission risks. Based on assuming unchanged 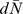, we used kernel *c = *5 and the dataset of the Dutch pig farm structure in 1997 and in 2011 to calculate the predicted change in the local between-farm CSF transmission risk across The Netherlands if also the other kernel parameters (λ_0_,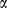 and 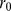) would remain unchanged. The local transmission risk is measured by the local reproduction ratio between farms [8], [14], and its change describes the net effect resulting from a lower farm density together with the increases in mean farm susceptibility and infectivity. The result of this net effect calculation indicates that the local CSF transmission risk pattern remains approximately the same in 2011 as it was in 1997: both the mean local transmission risk and the proportion of farms situated in high-risk areas remain approximately the same. For a visualisation of the high-risk areas in 1997 see Figure 1. As the analysis in this paper used the data from the 1997/1998 CSF epidemic, the estimated transmission kernel corresponds to the frequencies of the (indirect) contacts between pig farms during the epidemic as well as by the bio-security prevailing at pig farms at that time. Since then, there have been significant changes in pig farming in The Netherlands, and it is very well possible that the changes in pig farming may have also led to improved farm-level biosecurity. In addition, the management of indirect between-farm contact risks during an epidemic could at present times be realized more effectively than in 1997/1998. These changes may in principle affect the (parameters of the) transmission kernel, and our extrapolation should therefore be considered as indicative. The indication that the transmission risks remain roughly equal if contact frequencies and biosecurity were unchanged between 1997 and 2011 implies that if the ‘larger but fewer’ farms in reality come with substantial improvements of biosecurity and/or lowering of the frequency of indirect contacts between farms during an epidemic control phase as compared to 1997/1998, the net result would be a lowering of transmission risks.

An interesting question is to which extent our results for the farm-size dependence can be generalized to other pathogens in pigs and/or other livestock species. One issue of importance here is the possible difference between farm-size dependencies of transmission of epidemic versus endemic pathogens (cf. pig influenza). Concerning farm infectivity: as explained above the saturating tendency is likely to be related to properties of the process of detection (and subsequent culling) in the epidemic case. In the case of endemic pathogens, most often the detection is not followed by culling but by other control measures, and thus the farm-size dependency may be different for that reason. Another observation is that the types of between-farm contacts are different in the endemic case. For the exotic/epidemic case of CSF studied here, transport bans exclude or at least limit direct transmission (infected animals moving between farms). In contrast, direct transmission may play an important role in between-farm transmission of endemic pathogens, possibly influencing the farm-size dependence of the farm susceptibility and infectivity.
